# Deep Robust Moving Horizon Estimation for Nonlinear Multi-Rate Systems

**DOI:** 10.3390/s26061967

**Published:** 2026-03-21

**Authors:** Rusheng Wang, Songtao Wen, Bo Chen

**Affiliations:** 1 Department of Automation, Zhejiang University of Technology, Hangzhou 310023, China; rswang12@zjut.edu.cn (R.W.); wst2076859215@gmail.com (S.W.); 2 Zhejiang Key Laboratory of Intelligent Perception and Control for Complex Systems, Hangzhou 310023, China; 3 Joint Research and Development Center of Zhejiang University of Technology and Beijing United Information Technology Co., Ltd., Hangzhou 310023, China

**Keywords:** nonlinear multi-rate system, moving horizon estimation, deep learning, robust stability

## Abstract

In this paper, a moving horizon estimation (MHE)-based state estimation problem is studied for asynchronous multi-rate nonlinear systems. First, the asynchronous multi-rate system is transformed into a synchronous system at measurement sampling points through pseudo-measurement synchronization modeling. Secondly, a MHE strategy with a time-discounted quadratic objective is proposed. Under the detectability assumption, the exponential stability of the proposed MHE is established via the Lyapunov method, and the corresponding linear matrix inequality (LMI) constraints are derived. Moreover, to address the model mismatch after synchronization, a deep learning-based framework is proposed to approximate and learn the weighting parameters of the MHE. Then, barrier-function regularization is introduced to enforce the aforementioned LMI feasibility conditions, keeping the learned weights within the feasible region throughout training. Finally, the result is illustrated by a target tracking example.

## 1. Introduction

State estimation plays a vital role in modern control and perception systems, providing accurate estimates and unknown state of the system in applications such as cyber–physical security [1], unmanned systems [2], and robotics [3]. At present, most state estimation problems consider the situation of synchronous sampling of sensors. However, heterogeneous sensors tend to operate at different sampling frequencies, which makes the system have multi-rate characteristics. In particular, the actual system is mostly nonlinear, which further increases the complexity and challenge of the multi-rate system estimation.

### 1.1. Related Works

For the estimation problem in multi-rate systems, it is difficult to directly apply single-rate state estimation methods to solve estimation problems under asynchronous measurement models. Therefore, it is crucial to convert the asynchronous multi-rate systems into synchronous single-rate systems. At present, one of the main approaches to solving asynchronous estimation problems is to combine wavelet theory [4] with recursive multiscale modeling [5]. The core idea of this approach is to decompose signals into orthogonal components at different resolutions, enabling the modeling of stochastic phenomena across multiple scales. On this basis, for multiresolution multisensor systems with unknown sensor structures, an online projection operator estimation using generalized compactly supported wavelets and a recursive least-squares algorithm was proposed in [6]. In [7], sensor allocation in discrete-time teleoperation systems was investigated using a multi-rate sampling architecture and Krasovskii-based stability conditions. Moreover, the lifting technique has been employed to address various multi-rate estimation challenges. In [8], an $l_{2}$–$l_{\infty }$ filtering was proposed to solve the multi-rate estimation problem by using lifting technique. In [9], an optimal state estimation algorithm was proposed in the sense of linear minimum mean square error for a single-sensor multi-rate system. However, the augmented manner of lifting technique makes a high computational cost and weak real-time ability.

To overcome the limitation of lifting technique, the pseudo measurement method was developed to achieve synchronization in multi-rate systems. Based on this approach, a distributed matrix-weighted fusion estimation algorithm was proposed in [10], and a optimal sequential fusion filter with unreliable measurements and correlated noises was investigated in [11]. Consider the networked multi-rate systems, the variance-constrained state estimation problem was investigated in the presence of network-induced probabilistic sensor failures and measurement quantization [12], and a multi-rate $H_{\infty }$ filter was proposed for networked multisensor fusion systems with packet dropouts [13]. More recently, Sequential Student’s t-based UKF Fusion (SR-ASSTUKF) was proposed in [14] for nonlinear multi-rate multisensor systems with heavy-tailed noise and missing measurements, and it incorporated a *t*-test-driven adaptive mechanism to cope with nonstationary measurement uncertainty. However, it remains a one-step recursive fusion filter and does not explicitly optimize over a moving horizon to compensate for pseudo-measurement-induced model mismatch. Note that the above methods have not considered model mismatch in the synchronous modeling process, nor the need for adaptive weighting under time-varying noise conditions.

Different from conventional nonlinear filter that rely on one-step updates, such as extended Kalman filter (EKF) and unscented Kalman filter (UKF) [15], moving horizon estimation (MHE) optimizes state estimates over a finite moving window. Noted that the moving time window can better handle the asynchronous multi-rate measurements. In [16], the deviation from past estimates was penalized based on confidence weighting, where the arrival cost term in the MHE objective function was approximated by using an EKF-based covariance update. In [17], a suboptimal MHE was proposed that achieves robust stability not through optimization, but by constructing a feasible candidate solution. Additionally, a moving horizon estimator with robust global asymptotic stability was proposed in [18] under bounded disturbances. However, conventional MHE schemes generally neglect the effect of model mismatch, which undermines their robustness under strong nonlinearities and asynchronous measurements.

To improve estimation robustness, recent studies have incorporated deep learning into the MHE framework to compensate for the deficiencies of observation model under multi-rate conditions. In [19], a neural network-based nonlinear approximation function was introduced to improve estimation accuracy during the cost minimization process. Moreover, an autotuning NeuroMHE scheme was proposed in [20], where a multi-layer perceptron dynamically adjusts the weighting parameters of the estimation window. In [21], variational-Bayes adaptive MHE was proposed to guarantee mean-square boundedness under stochastic formulations. In [22], Gaussian-process-based MHE learned unknown dynamics with Gaussian-process regression and proved practical robust exponential stability with convergence to an error neighborhood. Furthermore, a recent systematic review was presented in [23], it shows that Neural MHE research covers model learning, cost learning, and optimization approximation, but rigorous stability guarantees beyond empirical validation are still widely missing. Noted that the above learning-based MHE studies typically establish theoretical guarantees in weaker senses, rather than in the robust global exponential stability (RGES) sense required for bounded-disturbance nonlinear systems. Obviously, the latter is more suitable for systems that contain time-varying learning-based components.

### 1.2. Motivations and Contributions

When the MHE weights are learned in a data-driven manner, the estimator is no longer a time-invariant optimization-based mapping. As a result, classical RGES analyses, which rely on uniform detectability/contractivity and uniformly positive bounded weights, do not apply directly. Without an explicit mechanism that constrains the learned weights to a stability-preserving region, learning may induce extreme or fast-varying weights, thereby violating these conditions under bounded disturbances and nonlinear dynamics. Recently, a proportional–integral filter has been proposed in [24] to handle the bounded disturbance under linear single-rate framework, and the boundedness of the Lyapunov–Krasovskii functional was also derived. On this basis, the nonfragile extended dissipativity state estimator was designed in [25] for discrete-time neural networks to ensure estimation performance under time-varying delays. In this case, how to effectively compensate for multi-rate observation errors using deep learning while preserving the robust stability structure of MHE remains a challenging problem.

Motivated by the above analysis, this work will investigate the deep learning-based robust MHE framework for addressing the estimation problem of nonlinear multi-rate systems. The main contributions are summarized as follows:

- A robust MHE is developed for nonlinear multi-rate systems, where the linear matrix inequality (LMI) constraints are derived to ensure the boundedness and exponential convergence of the estimation error under bounded disturbances.
- A stability-preserving neural framework is developed by embedding the LMI feasibility conditions into the learning process. This design keeps the adaptive weights within a feasible set and prevents learning-induced variations from undermining the required RGES conditions.

Notation: Let $R_{\geq 0}$ denote the positive real numbers and $I_{\geq 0}:=\left\{0,1,2,\ldots \right\}$ denote the set of non-negative integers. The identity matrix is denoted by $I_{n}\in R^{n\times n}$. For a vector $x\in R^{n}$, its Euclidean norm is denoted by ∥x∥. The notation det(·) denotes the determinant of a square matrix. Moreover, for any positive definite matrix $P=P^{⊤}$, the quadratic norm is defined by ${∥x∥}_{P}^{2}:=x^{⊤}Px$. Let $\lambda_{\max}\left(A,B\right):=\max_{x\neq 0}\frac{x^{⊤}Ax}{x^{⊤}Bx}$ denote the maximum generalized eigenvalue of positive definite matrices *A* and *B*. A function $\alpha :R_{\geq 0}\to R_{\geq 0}$ is of class K if it is continuous, strictly increasing, and satisfies α(0)=0. In addition, if α is unbounded, then $\alpha \in K_{\infty }$. A function $\beta :R_{\geq 0}\times I_{\geq 0}\to R_{\geq 0}$ is of class KL if β(·,t)∈K for each fixed $t\in I_{\geq 0}$ and β(r,·)∈L for each fixed $r\in R_{\geq 0}$. The term o(α) denotes a quantity such that $\lim_{\alpha \to 0}\frac{o(\alpha )}{\alpha }=0,$ i.e., o(α) is of higher order than α as α→0.

## 2. Problem Formulation

### 2.1. System Model

Consider the following asynchronous multi-rate nonlinear system with *m* sensors

(1) $$\begin{array}{cc} x_{t+1} & =f(x_{t},w_{t}) \end{array}$$

(2) $$\begin{array}{cc} y_{t}^{\left(i\right)} & =\phi^{\left(i\right)}\left(x_{t}\right)+v_{t}^{\left(i\right)}\;\mathrm{for}\;i=1,2,\ldots ,m \end{array}$$

where $x_{t}\in X\subseteq R^{n}$ is the system state, $y_{t}^{\left(i\right)}\in Y\subseteq R^{m_{i}}$ is the measurement collected by the *i*-th sensor. The disturbance input signal $w_{t}$ and measurement noise $v_{t}^{\left(i\right)}$ are locally essentially bounded functions taking values in set $W\subseteq R^{p}$ and $V^{\left(i\right)}\subseteq R^{q_{i}}$, respectively. The nonlinear mappings f:X×W→X and $\phi^{\left(i\right)}:X\times V\to Y$, which describe the system dynamics and output equation, are assumed to be jointly continuous. Meanwhile, X and W are closed.

Assume that the *i*-th sensor provides measurement $y_{t}^{\left(i\right)}$ every $T_{i}$ time unit, where $T_{i}$ is a positive integer multiple of the state updating period *T*. In this case, the asynchronous of the measurments will increase the design difficulty of the estimator. To synchronize the system state equation and measurement equation, a pseudo-measurement modeling approach [10] is adopted. Specifically, define a binary function:

(3) $$\theta_{i}\left(t\right)=\left\{\begin{array}{cc} 1, & \mathrm{if}\;\exists \;k\in I_{\geq 0}\;s.t.\;tT=k\;T_{i},\\ 0, & \mathrm{otherwise}, \end{array}\right.$$

where $\theta_{i}\left(t\right)=1$ denotes that a new measurement is available at time *t*; otherwise, the sensor is not sampled.

Subsequently, the predicted value is used as the pseudo-measurement for compensating sensor information at the unsampled moment, yielding the following reconstructed measurement model:

(4) $$y_{t}^{\left(i\right)}=\theta_{i}\left(t\right)\left[\phi^{\left(i\right)}\left(x_{t}\right)+v_{t}^{\left(i\right)}\right]+\left[1-\theta_{i}\left(t\right)\right]\phi^{\left(i\right)}\left({\bar{x}}_{t}\right)$$

where ${\bar{x}}_{t}$ is the predicted value of $x_{t}$. Let

(5) $$\begin{array}{cc} \theta (t) & :=\mathrm{diag}\left\{\theta_{1}\left(t\right),\ldots ,\theta_{m}\left(t\right)\right\}, \end{array}$$

(6) $$\begin{array}{cc} y_{t}^{⊤} & :=\left[\begin{array}{ccc} y_{t}^{\left(1\right)} & \ldots & y_{t}^{\left(m\right)} \end{array}\right], \end{array}$$

(7) $$\begin{array}{cc} \Phi^{⊤}\left(\cdot \right) & :=\left[\begin{array}{ccc} \phi^{\left(1\right)}\left(\cdot \right) & \ldots & \phi^{\left(m\right)}\left(\cdot \right) \end{array}\right], \end{array}$$

(8) $$\begin{array}{cc} v_{t}^{⊤} & :=\left[\begin{array}{ccc} v_{t}^{\left(1\right)} & \ldots & v_{t}^{\left(m\right)} \end{array}\right]. \end{array}$$

From (2)–(8), the reconstructed synchronous measurement model can be given by:

(9) $$\begin{array}{cc} y_{t} & =\theta \left(t\right)\left[\Phi \left(x_{t}\right)+v_{t}\right]+\left[I_{m}-\theta \left(t\right)\right]\Phi \left({\bar{x}}_{t}\right)\\ \; & =\theta \left(t\right)\Phi \left(x_{t}\right)+\left[I_{m}-\theta \left(t\right)\right]\Phi \left({\bar{x}}_{t}\right)+\theta \left(t\right)v_{t}\\ \; & :=h\left(x_{t}\right)+\theta \left(t\right)v_{t}. \end{array}$$

In this paper, the initial estimate ${\hat{x}}_{0}$ is used to moving estimate the state at time *t*, where ${\hat{x}}_{0}$ is unknown but belong to a known compact set X. Generally, MHE formulates state estimation as a finite-horizon optimization, where an arrival cost encodes past information, while a running cost penalizes process and measurement residuals. Thereby, based on the input–output history of system (1) and (9) over a sliding window with length $N:=\min\{N\in I_{>1},t\}$ at time *t*, the window-initial state ${\hat{x}}_{t-N|t}$ and disturbance sequence ${\hat{w}}_{\cdot |t}={\left\{{\hat{w}}_{j|t}\right\}}_{j=t-N}^{t-1}$ can be obtained by the MHE, and the corresponding estimate ${\hat{x}}_{\cdot |t}={\left\{{\hat{x}}_{j|t}\right\}}_{j=t-N}^{t-1}$ over the *N*-step horizon is generated by recursing system dynamic (1) and (9). It is concluded that the MHE formulates the following optimization problem:

(10) $$\begin{array}{cc} \min_{{\hat{x}}_{t-N|t},{\hat{w}}_{\cdot |t}}J & =2\eta^{N}{∥{\hat{x}}_{t-N|t}-{\hat{x}}_{t-N}∥}_{\bar{P}}^{2}\\ \; & +2\sum_{j=1}^{N}\eta^{j-1}∥{\hat{w}}_{t-j|t}{∥}_{Q}^{2}+2\sum_{j=0}^{N}\eta^{j}{∥h\left({\hat{x}}_{t-j|t}\right)-y_{t-j}∥}_{R}^{2},\\ \; & s.t.\;{\hat{x}}_{j+1|t}=f\left({\hat{x}}_{j|t},{\hat{w}}_{j|t}\right),\;j\in I_{\left[t-N,t-1\right]},\\ \; & {\hat{x}}_{\cdot |t}\in X,\;{\hat{w}}_{\cdot |t}\in W \end{array}$$

where η, *Q*, *R* and $\bar{P}$ are corresponding optimization parameters. Note that ${\hat{x}}_{t-N}$ denotes the arrival state estimate at the beginning of the current horizon, which is derived from the previous estimation result.

Denote ${\hat{x}}_{t-N|t}^{*}$ and ${\hat{w}}_{\cdot |t}^{*}$ as the optimal values of Problem  (10), and the estimate at time *t* can be obtained by propagating the optimal window-initial estimate:

(11) $${\hat{x}}_{t-N+1|t}^{*}=f\left({\hat{x}}_{t-N|t}^{*},{\hat{w}}_{t-N|t}^{*}\right).$$

It should be pointed out that the updated measurement sequence $\left\{y_{t-N+1},\ldots ,y_{t+1}\right\}$ is used to resolve the optimization problem (10) at time t+1, and then yielding a new estimate ${\hat{x}}_{t-N+1|t+1}^{*}$. This shift-and-update procedure is iteratively performed for all t≥N, thereby implementing the moving horizon estimation process in a receding-horizon manner.

### 2.2. Problem of Interest

Note that model synchronization renders the measurement information from (4) non-stationary, while traditional MHE with fixed weights is sensitive to this non-stationarity, and thus requiring frequent adjustment of the weights. To overcome this limitation, we will design an adaptive deep robust MHE (DRMHE).

To construct that the robust stability of the proposed DRMHE, an appropriate detectability assumption is required. Particularly, a suitable nonlinear detectability concept is incremental input/output-to-state stability (i-IOSS) [26]. In this case, the i-IOSS Lyapunov function has become a standard framework for describing nonlinear detectability [27,28,29].

**Definition** **1** (i-IOSS Lyapunov function [28])**.** *A function $V:X\times X\to R_{\geq 0}$ is an i-IOSS Lyapunov function for the system (1) and (9) if it is continuous, and there exist functions $\alpha_{1},\alpha_{2}\in K_{\infty }$, $\sigma_{w},\sigma_{v}\in K$ and a constant η∈[0,1) such that the following dissipation inequalities hold:*

(12) $$\begin{array}{cc} \alpha_{1}(∥x-\tilde{x}∥) & \leq V\left(x,\tilde{x}\right)\leq \alpha_{2}(∥x-\tilde{x}∥), \end{array}$$

(13) $$\begin{array}{cc} \;V(f\left(x,w\right),f\left(\tilde{x},\tilde{w}\right)) & \leq \;\eta V\left(x,\tilde{x}\right)+\sigma_{w}(∥w-\tilde{w}∥)+\sigma_{v}(∥y-\tilde{y}∥) \end{array}$$

*where $x,\tilde{x}\in X$, w∈W, $y,\tilde{y}\in Y$, and the nonlinear mapping relationship between $y,\tilde{y}$ and $x,\tilde{x}$ is derived from  (9).*

**Definition** **2** (RGES [30])**.** *A state estimator for system (1) and (9) is RGES if there exist functions β∈KL,$\pi_{w},\pi_{v}\in K$ and $\mu_{1},\mu_{2}\in \left[0,1\right)$ such that for all $x_{0},{\hat{x}}_{0}\in X$, w∈W, and v∈V the estimated state ${\hat{x}}_{t}$ satisfies*

(14) $$\begin{array}{cc} ∥x_{t}-{\hat{x}}_{t}∥\;\leq & \beta (∥x_{0}-{\hat{x}}_{0}∥,t)\\ \; & +\sum_{\tau =1}^{t}\mu_{1}^{\tau }\pi_{w}(∥w_{t-\tau }∥)+\sum_{\tau =0}^{t}\mu_{2}^{\tau }\pi_{v}(∥v_{t-\tau }∥). \end{array}$$

Detectability ensures the linear convergence of estimation errors within a finite horizon [30], which is a standard requirement for the robust exponential stability of MHE. Under the above definitions, the detectability assumption and regularity conditions are presented as follows.

**Assumption** **1** (Detectability [28])**.** *For each $t\in I_{\geq 0}$, system (1) and (9) admits a i-IOSS Lyapunov function V according to Definition 1 with quadratic bounds and supply rates, i.e., there exist $\underline{P},\bar{P}≻0$ and Q,R⪰0 such that*

(15) $$\begin{array}{cc} ∥x_{t}-{\tilde{x}}_{t}{∥}_{\underline{P}}^{2} & \leq V\left(x_{t},{\tilde{x}}_{t}\right)\leq {∥x_{t}-{\tilde{x}}_{t}∥}_{\bar{P}}^{2},\; \end{array}$$

(16) $$\begin{array}{cc} V(x_{t+1},{\tilde{x}}_{t+1}) & \leq \eta V\left(x_{t},{\tilde{x}}_{t}\right)+\;∥w_{t}-{\tilde{w}}_{t}{∥}_{Q}^{2}+\;{∥y_{t}-{\tilde{y}}_{t}∥}_{R}^{2},\; \end{array}$$

*for all $x_{t},{\tilde{x}}_{t}\in X$, $w_{t},{\tilde{w}}_{t}\in W$, and $y_{t},{\tilde{y}}_{t}\in Y$.*

**Assumption** **2.** *The function f is continuously differentiable in all of its arguments, h is affine in (x,w), and the sets X and W are convex.*

In conclusion, the problems to be solved in this paper are summarized as follows:

1. Under Assumption 1 and sensor asynchronous sampling, how may we prove the robust exponential stability of the proposed MHE and derive the corresponding LMI conditions?
2. Under Assumption 2, how may we employ deep learning to select time-varying weights within the admissible feasibility set without violating the LMI constraints?

## 3. Stability Analysis of Designed MHE

In order to establish RGES of the proposed moving horizon estimation, the following lemma is introduced. It indicates that the function *V* in Assumption 1 can be regarded as the *N*-step Lyapunov function of MHE.

**Lemma** **1.** When Assumption 1 holds, the Lyapunov function V with respect to optimal state estimate ${\hat{x}}_{t|t}^{*}$ satisfies

(17) $$\begin{array}{cc} V({\hat{x}}_{t|t}^{*},x_{t})\leq & \;4\eta^{N}\lambda_{\max}\left(\bar{P},\underline{P}\right)V\left({\hat{x}}_{t-N},x_{t-N}\right)\\ \; & +4\sum_{j=1}^{N}\eta^{j-1}∥w_{t-j}{∥}_{Q}^{2}+4\sum_{j=0}^{N}\eta^{j}{∥\theta \left(t-j\right)v_{t-j}∥}_{R}^{2}, \end{array}$$

*for all $t\in I_{\geq 0}$, $x,\hat{x}\in X$, w∈W, and v∈V.*

**Proof of Lemma** **1.** By solving optimization problem (10), the optimal solutions ${\left\{{\hat{x}}_{j|t}^{*}\right\}}_{j=t-N}^{t}$∈X and ${\left\{{\hat{w}}_{j|t}^{*}\right\}}_{j=t-N}^{t-1}\in W$ at time *t* can be obtained. By iterating inequality (16) *N* times and combining it with the Lyapunov-function upper bound (15) in Assumption 1, one obtains

(18) $$\begin{array}{cc} V({\hat{x}}_{t|t}^{*},x_{t}) & \leq \eta^{N}V\left({\hat{x}}_{t-N|t}^{*},x_{t-N}\right)\\ \; & +\sum_{j=1}^{N}\eta^{j-1}∥{\hat{w}}_{t-j|t}^{*}-w_{t-j}{∥}_{Q}^{2}+\sum_{j=0}^{N}\eta^{j}{∥{\hat{y}}_{t-j|t}^{*}-y_{t-j}∥}_{R}^{2}\\ \; & \overset{\left(15),(9\right)}{\leq }\eta^{N}∥{\hat{x}}_{t-N|t}^{*}-x_{t-N}{∥}_{\bar{P}}^{2}+\sum_{j=1}^{N}\eta^{j-1}{∥{\hat{w}}_{t-j|t}^{*}-w_{t-j}∥}_{Q}^{2}\\ \; & +\sum_{j=0}^{N}\eta^{j}{∥h\left({\hat{x}}_{t-j|t}^{*}\right)+\theta \left(t-j\right)v_{t-j}-y_{t-j}∥}_{R}^{2}. \end{array}$$

Next, by applying the Cauchy–Schwarz inequality to the term $∥{\hat{w}}_{t-j|t}^{*}-w_{t-j}{∥}_{Q}^{2}$, and the triangle inequality to the term $∥h\left({\hat{x}}_{t-j|t}^{*}\right)+\theta \left(t-j\right)v_{t-j}-y_{t-j}{∥}_{R}^{2}$, we obtain

(19) $$\begin{array}{cc} \; & ∥{\hat{w}}_{t-j|t}^{*}-w_{t-j}{∥}_{Q}^{2}\leq 2∥{\hat{w}}_{t-j|t}^{*}{∥}_{Q}^{2}+2{∥w_{t-j}∥}_{Q}^{2},\\ \; & ∥h\left({\hat{x}}_{t-j|t}^{*}\right)+\theta \left(t-j\right)v_{t-j}-y_{t-j}{∥}_{R}^{2} \end{array}$$

(20) $$\begin{array}{cc} \; & \leq 2∥h\left({\hat{x}}_{t-j|t}^{*}\right)-y_{t-j}{∥}_{R}^{2}+2{∥\theta \left(t-j\right)v_{t-j}∥}_{R}^{2}. \end{array}$$

Similarly, for the first term in (18), one has

(21) $$\begin{array}{cc} ∥{\hat{x}}_{t-N|t}^{*}-x_{t-N}{∥}_{\bar{P}}^{2}= & \;∥{\hat{x}}_{t-N|t}^{*}-{\hat{x}}_{t-N}+{\hat{x}}_{t-N}-x_{t-N}{∥}_{\bar{P}}^{2}\\ \; & \leq 2∥{\hat{x}}_{t-N|t}^{*}-{\hat{x}}_{t-N}{∥}_{\bar{P}}^{2}+2{∥{\hat{x}}_{t-N}-x_{t-N}∥}_{\bar{P}}^{2}. \end{array}$$

By substituting (19)–(21) into (18), and combining the cost function (10) which lead to

(22) $$\begin{array}{cc} V\left({\hat{x}}_{t|t}^{*},x_{t}\right)\overset{\left(10\right)}{\leq } & \;J^{*}\left({\hat{x}}_{t-N|t}^{*},{\hat{w}}_{t-j|t}^{*}\right)+2\eta^{N}{∥{\hat{x}}_{t-N}-x_{t-N}∥}_{\bar{P}}^{2}\\ \; & +2\sum_{j=1}^{N}\eta^{j-1}∥w_{t-j}{∥}_{Q}^{2}+2\sum_{j=0}^{N}\eta^{j}{∥\theta \left(t-j\right)v_{t-j}∥}_{R}^{2}. \end{array}$$

Since $J^{*}\left({\hat{x}}_{t-N|t}^{*},{\hat{w}}_{\cdot |t}^{*}\right)$ is the minimum of (10), for any feasible ${\tilde{x}}_{t-N}\in X$ and ${\tilde{w}}_{\cdot |t}\in W$ it holds that

(23) $$\begin{array}{c} J^{*}\left({\hat{x}}_{t-N|t}^{*},{\hat{w}}_{\cdot |t}^{*}\right)\;\leq \;J\left({\tilde{x}}_{t-N},{\tilde{w}}_{\cdot |t}\right). \end{array}$$

Substituting this bound into the previous inequality (22) yields the desired estimate. Evidently, the true state and disturbance sequences, as a feasible solution to the MHE problem, also satisfy the above relationship. Hence, we obtain

(24) $$\begin{array}{cc} V({\hat{x}}_{t|t}^{*},x_{t})\leq & \;4\eta^{N}{∥{\hat{x}}_{t-N}-x_{t-N}∥}_{\bar{P}}^{2}\\ \; & +4\sum_{j=1}^{N}\eta^{j-1}∥w_{t-j}{∥}_{Q}^{2}+4\sum_{j=0}^{N}\eta^{j}{∥\theta \left(t-j\right)v_{t-j}∥}_{R}^{2}. \end{array}$$

Then, by utilizing the properties of the maximum generalized eigenvalue to reformulate the arrival cost, one has

(25) $$\begin{array}{cc} ∥{\hat{x}}_{t-N}-x_{t-N}{∥}_{\bar{P}}^{2} & \leq \lambda_{\max}\left(\bar{P},\underline{P}\right){∥{\hat{x}}_{t-N}-x_{t-N}∥}_{\underline{P}}^{2}\\ \; & \overset{\left(15\right)}{\leq }\lambda_{\max}\left(\bar{P},\underline{P}\right)V\left({\hat{x}}_{t-N},x_{t-N}\right), \end{array}$$

where $\lambda_{\max}\left(\bar{P},\underline{P}\right)$ denotes the maximum generalized eigenvalue of $\bar{P}$ and $\underline{P}$. By substituting (25) into (24), the relationship between the Lyapunov function at the initial time step and the current time step of the receding horizon is obtained.    □

Based on the *N*-step Lyapunov inequality (17) under Assumption 1, the RGES of the MHE will be analyzed below, with 

(26) $$\delta :=4\eta^{N}\lambda_{\max}\left(\bar{P},\underline{P}\right).$$

**Theorem** **1.** *When the Assumption 1 holds, there exists a horizon $N\in I_{>1}$ that is selected to satisfy 0≤δ<1, such that the proposed MHE estimator is RGES in the sense of Definition 2 for all $t\in I_{\geq 0}$.*

**Proof of Theorem** **1.** Define k:=⌊t/N⌋ and l:=tmodN for all $t\in I_{\geq 0}$, such that *t* can be expressed as t=kN+l. Then, the Lyapunov relationship under full information for t<N can be derived from (24):

(27) $$\begin{array}{cc} V\left({\hat{x}}_{t},x_{t}\right)=V\left({\hat{x}}_{l},x_{l}\right) & \leq 4\eta^{N}{∥{\hat{x}}_{0}-x_{0}∥}_{\bar{P}}^{2}\\ \; & +4\sum_{j=1}^{l}\eta^{j-1}∥w_{l-j}{∥}_{Q}^{2}+4\sum_{j=0}^{l}\eta^{j}{∥\theta \left(l-j\right)v_{l-j}∥}_{R}^{2}. \end{array}$$

When t≥N, applying the lower bound mentioned in Lemma 1 *k* times yields

(28) $$\begin{array}{cc} \; & V({\hat{x}}_{t},x_{t})\\ \; & \leq \delta^{k}V\left({\hat{x}}_{t-kN},x_{t-kN}\right)+4\sum_{i=0}^{k-1}\delta^{i}\sum_{j=1}^{N}\eta^{j-1}{∥w_{t-iN-j}∥}_{Q}^{2}\\ \; & +4\sum_{i=0}^{k-1}\delta^{i}\sum_{j=0}^{N}\eta^{j}{∥\theta \left(t-iN-j\right)v_{t-iN-j}∥}_{R}^{2}. \end{array}$$

Using the bound

(29) $$\begin{array}{c} ∥\theta \left(t\right)v_{t}{∥}_{R}^{2}\leq {∥v_{t}∥}_{R}^{2},\;\forall t\in I_{\geq 0} \end{array}$$

together with η≤δ, inequality (28) can be derived as

(30) $$\begin{array}{cc} V({\hat{x}}_{t},x_{t})\leq & \;4\delta^{t}{∥{\hat{x}}_{0}-x_{0}∥}_{\bar{P}}^{2}\\ \; & +4\sum_{\tau =1}^{t}\delta^{\tau }∥w_{t-\tau }{∥}_{Q}^{2}+4\sum_{\tau =0}^{t}\delta^{\tau }{∥v_{t-\tau }∥}_{R}^{2}. \end{array}$$

From (30), taking the square root of the lower bound in (15), we have

(31) $$\begin{array}{cc} ∥{\hat{x}}_{t}-x_{t}{∥}_{\underline{P}}\leq & \;\sqrt{V({\hat{x}}_{t},x_{t})}\leq 2\delta^{\frac{t}{2}}{∥{\hat{x}}_{0}-x_{0}∥}_{\bar{P}}\\ \; & +2\sum_{\tau =1}^{t}\delta^{\frac{\tau }{2}}∥w_{t-\tau }{∥}_{Q}+2\sum_{\tau =0}^{t}\delta^{\frac{\tau }{2}}{∥v_{t-\tau }∥}_{R}, \end{array}$$

where $\beta :=\delta^{\frac{t}{2}}{∥{\hat{x}}_{0}-x_{0}∥}_{\bar{P}}\in \mathrm{KL}$, $\pi_{w}:={∥w_{t-\tau }∥}_{Q}\in K$,$\pi_{v}:={∥v_{t-\tau }∥}_{R}\in K$, and $\mu_{1}=\mu_{2}:=\delta^{\frac{\tau }{2}}$. It is concluded from (31) that the estimation error satisfies (14) and the RGES of proposed MHE has been established.    □

On the basis of Theorem 1, the existence condition of i-IOSS Lyapunov function is provided in the following Theorem. In particular, the quadratic i-IOSS Lyapunov function can be certified via contraction/Riemannian methods [28].

Define the Jacobian linearizations of (1) and (9) at the operating point $(x_{t},w_{t},v_{t})\in X\times W\times V$ as

(32) $$\begin{array}{c} A_{t}=\frac{\partial f}{\partial x}{|}_{\left(x_{t},w_{t}\right)},\;\;B_{t}=\frac{\partial f}{\partial w}{|}_{\left(x_{t},w_{t}\right)},\\ C_{t}=\frac{\partial h}{\partial x}{|}_{\left(x_{t},v_{t}\right)},\;\;D_{t}=\frac{\partial \left(\theta \left(t\right)\;v_{t}\right)}{\partial v_{t}}{|}_{\left(x_{t},v_{t}\right)}. \end{array}$$

**Theorem** **2.** *When the Assumption 2 holds, if there exist symmetric matrices P≻0, Q,R⪰0 and a constant η∈[0,1) such that*

(33) $$\begin{array}{c} \left[\begin{array}{cc} A_{t}^{⊤}PA_{t}-\eta P-C_{t}^{⊤}RC_{t} & A_{t}^{⊤}PB_{t}-C_{t}^{⊤}RD_{t}\\ B_{t}^{⊤}PA_{t}-D_{t}^{⊤}RC_{t} & B_{t}^{⊤}PB_{t}-D_{t}^{⊤}RD_{t}-Q \end{array}\right]⪯0 \end{array}$$

*for all $(x_{t},w_{t},v_{t})\in X\times W\times V$. Then, $V\left(x,\tilde{x}\right)={∥x-\tilde{x}∥}_{P}^{2}$ is a i-IOSS Lyapunov function and satisfies Assumption 1 with $\bar{P}=\underline{P}=P$.*

**Proof of Theorem** **2.** See ([28], Theorem 2), the detailed proof is omitted.    □

Therefore, the LMI constraints are expressed as matrix inequalities in (33), which are crucial for ensuring the robustness and convergence of the MHE. In particular, they provide a verifiable sufficient condition that enforces the i-IOSS-based dissipation/contraction structure required by the RGES-type analysis.

**Remark** **1.** *Notice that the cost function J of MHE can be parameterized by any positive definite matrices P, Q, R and any η∈[0,1) when Assumption 2 holds. Moreover, if the uniform boundary is known a priori, the time-varying weights $P_{t}$, $Q_{t}$, $R_{t}$ can be determined by (10), and the detailed selection strategy will be presented in Section 4.*

## 4. Deep Robust MHE Based on Barrier Function

### 4.1. Architecture

Generally, the arrival cost covariance *P* is approximated via the EKF, which depends on the Jacobian matrices (32). However, the accuracy of the Jacobian matrices is affected by the pseudo measurement (4), and observation errors make the choice of *R* is crucial. To address this, a deep neural network [20] is adopted to regress the time-varying matrices $P_{t},Q_{t},R_{t}$.

Let $G:=\left[\begin{array}{c} P_{t}\;Q_{t}\;R_{t} \end{array}\right]$ denote the output of a residual network (ResNet) [31], and defining ${𝘍}_{\omega }$ as the ResNet with parameters ω, DRMHE assigns the weights via

(34) $$G\left(\omega \right):={𝘍}_{\omega }\left({\bar{x}}_{t}\right),$$

where ${\bar{x}}_{t}$ is the predicted state.

The estimated state $\hat{x}:={\hat{x}}_{\cdot |t}^{G}$ implicitly depends on G(ω), and thus on the network parameters ω. Specifically, this implicit dependence allows for the end-to-end learning of the parameters ω through gradient-based optimization.

Then, in order to obtain the process of learning the optimal parameter ω with respect to ResNet-based DRMHE, we formulate the following optimization problem:

(35) $$\begin{array}{cc} \; & \min_{\omega }\;L\left(G\left(\omega \right)\right)\\ \; & s.t.\;G(\omega )\;\mathrm{satisfies}\;(\mathrm{33}). \end{array}$$

It is noted that the loss in terms of estimation error over the horizon *N* is proposed in this paper, rather than the error between the neural network outputs and the ideal covariance. This is because the ideal covariance cannot be accurate determined using the model (1) and (9). In this case, the loss function is set to

(36) $$L_{track}\left(\hat{x}\right)=\frac{1}{N}\sum_{j=0}^{N}{∥{\hat{x}}_{t-j|t}^{G}-x_{t-j}∥}^{2},$$

where the gradient of the loss (36) with respect to the ResNet parameters ω is computed by backpropagation [32], and the chain rule is given by:

(37) $$\frac{dL_{track}}{d\omega }=\frac{\partial L_{track}\left(\hat{x}\right)}{\partial \hat{x}}\frac{\partial \hat{x}\left(G\right)}{\partial G}\frac{\partial G(\omega )}{\partial \omega }.$$

In fact, the training structure of ω is a bi-level optimization scheme. In the outer forward pass, the MHE optimizer receives G and generates the optimal solution $\hat{x}$, yielding the loss $L_{\mathrm{track}}$ In the backward pass, the gradient components $\frac{\partial L(\hat{x})}{\partial \hat{x}},\frac{\partial \hat{x}\left(G\right)}{\partial G}$ are computed and fed back to the network. Note that the gradient $\frac{\partial \hat{x}\left(G\right)}{\partial G}$ is recursively computed using a Kalman filter-based gradient solver [33]. The inner optimization computes $\frac{\partial G(\omega )}{\partial \omega }$ by standard machine learning tools, enabling the neural network to update G accordingly. In particular, the loss used for updating the neural network parameters is not the tracking error loss $L_{\mathrm{track}}$ itself, but rather its projected form:

(38) $$L_{\mathrm{neuro}}\left(\omega \right):={\left.\frac{\partial L_{\mathrm{track}}\left(\hat{x}\right)}{\partial \hat{x}}\right|}_{\hat{x}}\cdot {\left.\frac{\partial \hat{x}\left(G\right)}{\partial G}\right|}_{G}\cdot G\left(\omega \right).$$

It reflects the dependency of the final loss on the neural network parameters through the weighting map G.

### 4.2. Conflict Between Learning and RGES Guarantees

In learning-based weight adaptation, the weights are generated by

(39) $$G_{t}\left(\omega \right):={𝘍}_{\omega }\left(\xi_{t}\right),$$

where ω is fixed after offline training while the input $\xi_{t}$ varies over time, so the LMI constraints become time-varying through $\xi_{t}$. This reveals a structural conflict between RGES and unconstrained learning. RGES relies on the LMI/i-IOSS inequalities (33) holding for all time instants with time-uniform margins, so that time-uniform contraction constants and i-IOSS Lyapunov function exist. By contrast, $L_{\mathrm{neuro}}$ optimizes an average data-fitting objective and does not enforce (33). Therefore, the learned weights may violate the LMI constraints at some *t*, which breaks the uniform contraction argument and prevents concluding RGES.

To formalize this point, we model the time-varying LMI constraints as a scalar-valued constraint function

(40) $$c_{t}:R^{d}\to R,\;c_{t}\left(\omega \right)\leq 0,$$

where $c_{t}\left(\omega \right)\leq 0$ represents the satisfaction of the LMI constraints induced by the learned weights G(ω). For instance, $c_{t}\left(\omega \right)$ may be chosen as the maximum eigenvalue of the symmetric matrix defining the LMI residual at time *t*, but the following discussion only requires continuity and differentiability.

Define the LMI-constraints violation set at time *t* as

(41) $$D_{t}:=\left\{\omega \in R^{d}:\;c_{t}\left(\omega \right)>0\right\}.$$

If $c_{t}$ is continuous in ω, then $D_{t}$ is open in $R^{d}$.

**Assumption** **3.** *Assume that, for each time t, the scalar-valued constraint function $c_{t}\left(\cdot \right)$ is continuous and differentiable with respect to ω. Moreover, there exist a time $t^{★}$ such that*

(42) $$c_{t^{★}}\left(\omega^{★}\right)=0,\;\nabla c_{t^{★}}\left(\omega^{★}\right)\neq 0,$$

*where $\omega^{★}$ is a boundary point of the feasible set.*

**Corollary** **1.** *Consider an offline-trained parameter update driven by the neuro loss $L_{\mathrm{neuro}}$:*

(43) $$\omega^{+}=\omega -\alpha \;\nabla L_{\mathrm{neuro}}\left(\omega \right),$$

*with step size α>0. Suppose Assumption 3 holds. If exist some time t and a boundary point $\omega^{★}$ satisfying*

(44) $$c_{t}\left(\omega^{★}\right)=0,\;\nabla c_{t}\left(\omega^{★}\right)\neq 0,$$

*and the loss descent direction is misaligned with the LMI-feasible direction at $\omega^{★}$, i.e.,*

(45) $$\langle \nabla c_{t}\left(\omega^{★}\right),\;\nabla L_{\mathrm{neuro}}\left(\omega^{★}\right)\rangle <0.$$

*Then, there exists $\bar{\alpha }>0$ such that for all $\alpha \in (0,\bar{\alpha })$ the unconstrained update (43) violates the time-t LMI constraints, i.e., $\omega^{+}\in D_{t}$.*

**Proof.**  When Assumption 3 holds, a first-order Taylor expansion of $c_{t^{★}}$ at time $t^{★}$ along the update direction in (43) gives

(46) $$c_{t^{★}}\left(\omega^{+}\right)=c_{t^{★}}\left(\omega^{★}-\alpha \nabla L_{\mathrm{neuro}}\left(\omega^{★}\right)\right)=c_{t^{★}}\left(\omega^{★}\right)-\alpha \langle \nabla c_{t^{★}}\left(\omega^{★}\right),\nabla L_{\mathrm{neuro}}\left(\omega^{★}\right)\rangle +o\left(\alpha \right).$$

Using (44) and (45), the leading term satisfies

(47) $$-\alpha \langle \nabla c_{t^{★}}\left(\omega^{★}\right),\nabla L_{\mathrm{neuro}}\left(\omega^{★}\right)\rangle >0.$$

Hence, there exists some time *t* and $\bar{\alpha }>0$ such that $c_{t}\left(\omega^{+}\right)>0$ for all $\alpha \in (0,\bar{\alpha })$, which is equivalent to $\omega^{+}\in D_{t}$ by the definition of $D_{t}$.    □

**Remark** **2.** *Corollary 1 implies that once an unconstrained learning update yields a parameter ω such that $\omega \in D_{t}$ for some time t, the LMI constraints used to derive the N-step Lyapunov recursion may fail at that instant. As a consequence, the key inequality required to propagate a time-uniform contraction factor can no longer be guaranteed, and and the estimator may fail to satisfy RGES. This observation directly motivates the next subsection: we enforce $c_{t}\left(\omega \right)\leq 0$ during training via barrier-based constrained optimization, so that the LMI constraints remain satisfied for all relevant t and the RGES proof chain can be closed.*

### 4.3. Barrier-Based Optimization for Deep Robust MHE

This section addresses a critical challenge in DRMHE: enforcing strict satisfaction of LMI constraints during neural network training. However, the LMI constraints represent hard constraints incompatible with standard backpropagation. In this case, a barrier-based optimization method is proposed, which integrates barrier regularization with the tracking error loss $L_{\mathrm{track}}$ to ensure neural network weights satisfy i-IOSS conditions.

According to the negative semi-definiteness of matrix inequalities, an equivalent Schur complement form of (33) is derived:

(48) $$\begin{array}{cc} \; & \;T\left(\omega \right):=A_{t}^{⊤}PA_{t}-\eta P-C_{t}^{⊤}R_{t}C_{t}⪯0,\\ \; & \;S\left(\omega \right):=B_{t}^{⊤}PB_{t}-D_{t}^{⊤}R_{t}D_{t}-Q-\left(B_{t}^{⊤}PA_{t}-D_{t}^{⊤}R_{t}C_{t}\right) \end{array}$$

(49) $$\begin{array}{cc} \; & {\left(A_{t}^{⊤}PA_{t}-\eta P-C_{t}^{⊤}R_{t}C_{t}\right)}^{-1}\left(A_{t}^{⊤}PB_{t}-C_{t}^{⊤}R_{t}D_{t}\right)⪯0. \end{array}$$

The hard constraint (48) and (49) are incorporated into the loss function via a barrier function:

(50) $$\begin{array}{cc} g_{1}\left(\omega \right) & =-\ln\det\left(-T\left(\omega \right)-\varepsilon I_{n}\right), \end{array}$$

(51) $$\begin{array}{cc} g_{2}\left(\omega \right) & =-\ln\det\left(-T\left(\omega \right)-\varepsilon I_{p}\right), \end{array}$$

with a small margin ε>0.

Hence, we modify $L_{\mathrm{neuro}}$ to

(52) $$\begin{array}{cc} L_{neuro}\left(\omega \right)= & \;\frac{\partial L_{track}\left(\hat{x}\right)}{\partial \hat{x}}{|}_{\hat{x}}\cdot \frac{\partial \hat{x}\left(G\right)}{\partial G}{|}_{G}\cdot G\left(\omega \right)\\ \; & +\kappa \cdot {∥\omega ∥}^{2}+\gamma \cdot \sum_{i=1}^{2}g_{i}\left(\omega \right), \end{array}$$

where κ>0 is a regularization coefficient and γ>0 is a penalty factor. Minimize the loss metric (52) by training the weights G to enhance the robustness of MHE, thereby improving the state estimation performance.

The resulting procedure of bi-level optimization is summarized as Algorithm 1.
**Algorithm 1** Bi-level Optimization for Deep Robust MHE**Input:** Predicted state ${\bar{x}}_{t}$;**Initialization:** Initial tuning parameter $\omega_{0}$;**for** i=1 to $N_{\mathrm{epochs}}$ **do**    **for** t=0 to *T* **do**       **Outer Optimization (Forward Pass):**      Solve the MHE problem (10) by substituting G(ω) to obtain $\hat{x}$;       Compute batch loss $L_{track}$ using (36);       **Outer Optimization (Backward Pass):**       Compute gradient components $\frac{\partial L_{track}\left(\hat{x}\right)}{\partial \hat{x}}$, $\frac{\partial \hat{x}\left(G\right)}{\partial G}$;       **Inner Optimization (neural network):**       Compute $\frac{\partial L_{neuro}\left(\omega \right)}{\partial \omega }$ using machine learning tool;       Update ω using gradient-based optimization;       Update adaptive weightings G(ω) using (34);    **end for****end for****return** ω.

## 5. Numerical Example

Consider a vehicle localization system in a two-dimensional horizontal space, where three sensors are used to track the state of the target. The nonlinear vehicle motion model can be given by [34]:

(53) $$\begin{array}{cc} x_{t+1}^{p} & =x_{t}^{p}+\Delta t\cdot \nu \cos\left(\psi_{t}+\beta_{t}\right),\\ y_{t+1}^{p} & =y_{t}^{p}+\Delta t\cdot \nu \sin\left(\psi_{t}+\beta_{t}\right),\\ \psi_{t+1} & =\psi_{t}+\Delta t\cdot \frac{\nu }{l_{r}}\sin\left(\beta_{t+1}\right),\\ \beta_{t} & =\tan^{-1}\left(\frac{l_{r}}{l_{r}+l_{f}}\tan\left(c_{t}\right)\right), \end{array}$$

where $x_{t+1}^{p}$ and $y_{t+1}^{p}$ are the positions of the vehicle along the X-axes and Y-axes, respectively, $\psi_{t}$ denotes the heading angle, ν is the constant speed. The parameter Δt denotes the sampling time, $l_{r}$ and $l_{f}$ denote the distances to the rear and front axles, respectively. The side-slip angle $\beta_{t}$ is determined by the control input $c_{t}$. According to (53), the nonlinear state equation can be expressed by

(54) $$f\left(x_{t},w_{t}\right)≜\left[\begin{array}{c} x_{t}^{p}+\Delta t\cdot \nu \cos\left(\psi_{t}+\beta_{t}\right)\\ y_{t}^{p}+\Delta t\cdot \nu \sin\left(\psi_{t}+\beta_{t}\right)\\ \psi_{t}+\Delta t\cdot \frac{\nu }{l_{r}}\sin\left(\beta_{t}\right) \end{array}\right]+\left[\begin{array}{c} w_{t}^{x}\\ w_{t}^{y}\\ w_{t}^{\psi } \end{array}\right].$$

Moreover, three sensors indexed by i=1,2,3 observe the target, providing heading angle $\psi_{t}$ and planar position components $\left(x_{t}^{p,(i)},y_{t}^{p,(i)}\right)$. Meanwhile, the measurement equations are same as (2). In detail, the simulation setup of initial state is set to $x_{0}={\left[-0.5,-0.5,\pi /4\right]}^{⊤}$, constant speed ν=3, front/rear axle lengths $l_{r}=l_{f}=0.5$, and control input $c_{t}=5.73^{\circ }$. The sampling time is Δt=0.2, and sensor update intervals are 0.2, 0.3, and 0.6, respectively. The MHE horizon and discount factor are N=10 and η=0.4. Moreover, the process and measurement noise variances are $\sigma_{p}^{2}=10^{-2}$ and $\sigma_{m}^{2}=10^{-3}$.

**Figure 1:**
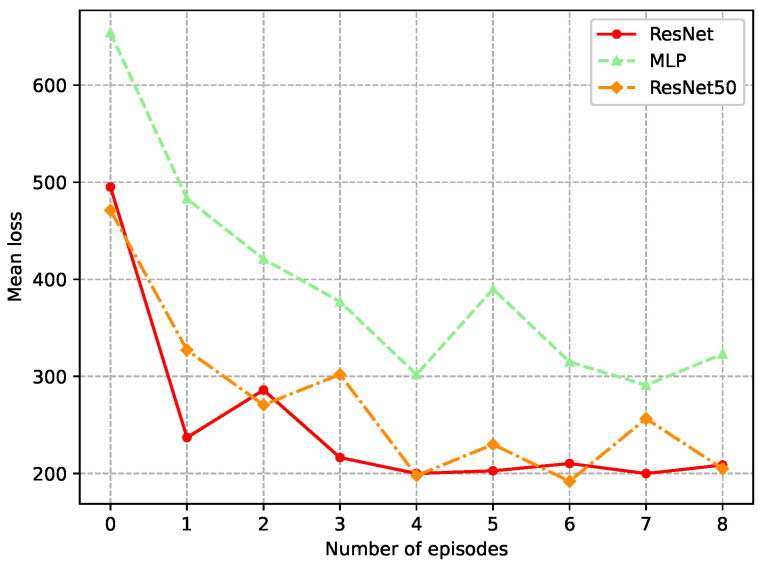
Mean training loss per episode for the DRMHE weight network.

The neural network is implemented as a ResNet with a block configuration of [2, 3, 4, 2] and Rectified Linear Unit (ReLU) activations. It takes the predicted state ${\bar{x}}_{t}$ as input, and employs residual connections to alleviate the vanishing-gradient problem. The training set consists of time steps 100–5000, and testing is performed on a separate trajectory generated under different initial conditions and noise. This multi-rate simulation with bounded noise and bounded disturbances is adopted to ensure controlled and repeatable evaluation, and it is consistent with the assumptions required for the RGES guarantee. The Adam optimizer is adopted for training, and the mean loss is compared in Figure 1 across different network architectures, including a standard ResNet, ResNet50 with deeper residual block configurations, and a multilayer perceptron (MLP). It can be observed that the ResNet network achieves a lower mean loss than the MLP and reaches a stable level more rapidly. In contrast, ResNet50 shows less smooth training loss and overfitting, which is caused by its excessively deep network structure. Based on the definition of the loss function (52), it is indicated that the DRMHE framework using ResNet exhibits excellent convergence and training efficiency.

**Figure 2:**
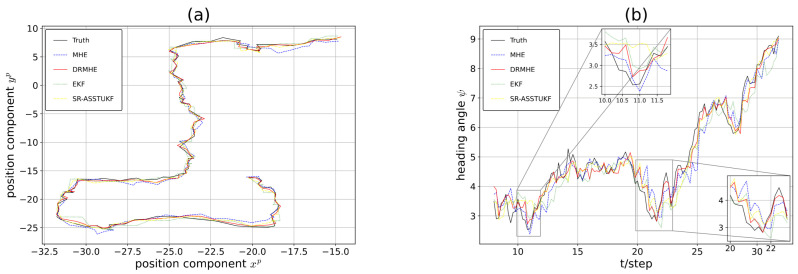
(**a**) Position components in the $x^{p}$ and $y^{p}$ directions. (**b**) Estimation trajectories for the heading angle ψ.

The true trajectory and the tracking trajectories of DRMHE, MHE, EKF, and SR-ASSTUKF [14] are plotted in Figure 2. The inset in Figure 2b shows the estimated heading angle at the moment of fastest change. EKF and MHE exhibit limited robustness, whereas SR-ASSTUKF achieves improved tracking via Student’s t-based sequential fusion. Nevertheless, because the yaw angle is measured at the lowest rate and experiences the most frequent dropouts, the robustness of SR-ASSTUKF decreases as measurement missingness becomes more severe. It demonstrates that DRMHE reacts more quickly, exhibits lower latency, and has smaller error spikes, enabling it to return to the normal trend faster than other methods.

**Figure 3:**
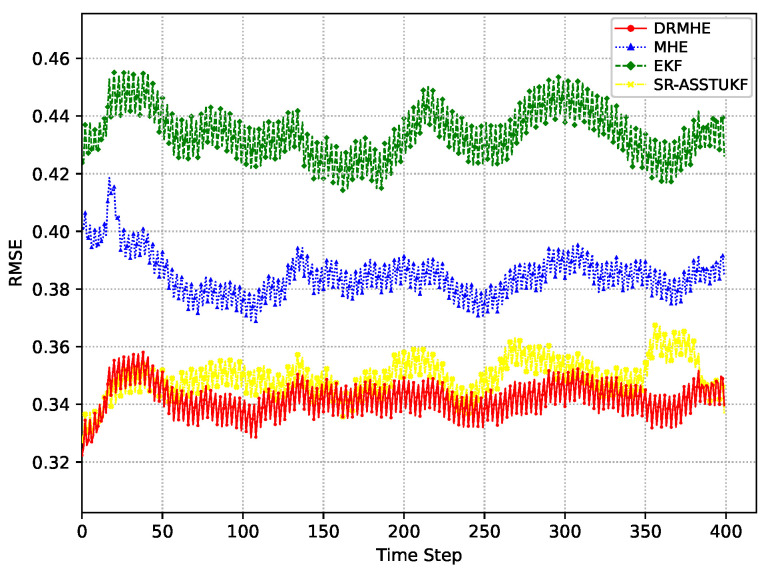
Estimation performance comparison of DRMHE, SR-ASSTUKF, MHE, and EKF.

Meanwhile, the root mean square error (RMSE) is adopted to evaluate the estimation performance of multisensor multi-rate estimators. In particular, to mitigate occasional outcomes caused by random noise in a single run, 200 Monte Carlo simulation runs are conducted. The RMSE of these estimator are reported to ensure statistical significance in Figure 3. It can be observed that the RMSE of the proposed DRMHE is lower than those of EKF [15], MHE [28], and SR-ASSTUKF [14], indicating superior estimation accuracy of DRMHE. It shows the DRMHE exhibits only slight fluctuations while maintaining a low estimation error, and indicates superior robustness and stability of proposed DRMHE under interference and asynchronous sampling. It can be further observed that the RMSE of DRMHE decays more rapidly and reaches its steady level earlier. Moreover, a numerical table of RMSEs of different methods is presented in Table 1, which shows DRMHE has a lower tracking error intuitively.

**Figure 4:**
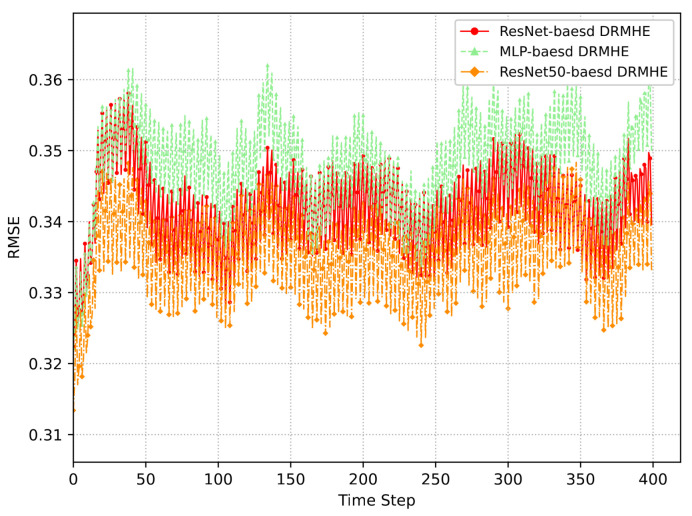
Estimation performance comparison of DRMHE based on ResNet, ResNet50 and MLP.

Figure 4 compares the RMSEs of MLP-based DRMHE, ResNet-based DRMHE, and ResNet50-based DRMHE. Meanwhile, the computational time required for DRMHE using these different network structures under 40 simulation experiments is reported in Table 2. It can be seen that ResNet achieves superior estimation performance compared with MLP, while the deeper ResNet50 network results in less smooth convergence and increased computational cost. This performance pattern is mainly attributed to the fact that the learning algorithm in this study is applied to multi-sensor data with a relatively small dataset, indicating that the depth of ResNet should be adjusted to the amount of data required.

## 6. Conclusions

In this paper, a deep robust moving horizon estimation method for nonlinear multi-rate systems was proposed. In detail, a pseudo-measurement compensation mechanism was designed to establish a synchronization model for multi-rate systems. Under the detectability assumption, Lyapunov-based analysis was employed to derive the LMI constraints for ensuring RGES of the estimation framework. To reduce the impact of model mismatch caused by pseudo-measurements, ResNet was employed to approximate the weighting parameters, thereby improving estimation robustness and accuracy. Then, a barrier-regularized training strategy was designed to reconcile the structural conflict between neural weight learning and RGES guarantees, by restricting the learned weights to a feasible region throughout training. Finally, a simulation example was presented to show the effectiveness of the proposed algorithm.

This paper considered a multirate systems with sensor uniform sampling, while the complexity of the real environment leads to many non-uniform sampling situations for sensors. Meanwhile, MHE scheme essentially has an insufficiency in terms of computational efficiency. In this case, the non-uniform sampling of sensors will be studied, and the learning-based nonlinear fusion criterion will be further designed to adapt to the effective fusion of asynchronous multimodal information. To reduce computational burden, future work will study stability guarantees for suboptimal MHE under a fixed number of solver iterations or early-termination rules, while preserving RGES-type properties as much as possible. Moreover, how to extend the framework to networked systems with packet dropouts and limited communication resources also be our future work.

## Figures and Tables

**Table 1:** The RMSEs of the tracking errors of DRMHE, EKF, MHE and SR-ASSTUKF methods, under bounded noise, bounded perturbation and asynchronous sampling.

Method	DRMHE	EKF	MHE	SR-ASSTUKF
RMSE	0.342	0.433	0.384	0.349

**Table 2:** The computational time required for DRMHE based on ResNet, ResNet50 and MLP.

Network	ResNet	ResNet50	MLP
Run time/ms	14.854	22.887	10.506

## Data Availability

The original contributions presented in this study are included in the article. Further inquiries can be directed to the corresponding author.
